# The repellency of lemongrass oil against stable flies, tested using video tracking

**DOI:** 10.1051/parasite/2013021

**Published:** 2013-06-13

**Authors:** Frédéric Baldacchino, Coline Tramut, Ali Salem, Emmanuel Liénard, Emilie Delétré, Michel Franc, Thibaud Martin, Gérard Duvallet, Pierre Jay-Robert

**Affiliations:** 1 Dynamique et Gouvernance des Systèmes Écologiques, Centre d’Écologie Fonctionnelle et Évolutive (CEFE), UMR 5175, Université Paul-Valéry (UM3) Montpellier France; 2 Laboratoire de Parasitologie, École Nationale Vétérinaire (ENVT) Toulouse France; 3 Centre de Coopération Internationale en Recherche Agronomique pour le Développement (CIRAD), UR-Hortsys Montpellier France

**Keywords:** *Stomoxys calcitrans*, stable fly, repellent, lemongrass, *Cymbopogon citratus*, video tracking

## Abstract

Lemongrass oil (*Cymbopogon citratus*) is an effective repellent against mosquitoes (Diptera: Culicidae) and house flies (Diptera: Muscidae). In this study, its effectiveness was assessed on stable flies (Diptera: Muscidae) in laboratory conditions. First, we demonstrated that lemongrass oil is an active substance for antennal olfactory receptor cells of *Stomoxys calcitrans* as indicated by a significant increase in the electroantennogram responses to increasing doses of lemongrass oil. Feeding-choice tests in a flight cage with stable flies having access to two blood-soaked sanitary pads, one of which was treated with lemongrass oil, showed that stable flies (*n* = 24) spent significantly more time in the untreated zone (median value = 218.4 s) than in the treated zone (median value = 63.7 s). No stable flies fed on the treated pad, whereas nine fed on the untreated pad. These results suggest that lemongrass oil could be used as an effective repellent against stable flies. Additional studies to confirm its spatial repellent and feeding deterrent effects are warranted.

## Abstract (fr)

L’huile essentielle de *Cymbopogon citratus* est un répulsif actif contre les moustiques (Diptera : Culicidae) et les mouches domestiques (Diptera : Muscidae). Dans cette étude, nous avons testé son efficacité contre les stomoxes (Diptera : Muscidae) en laboratoire. Nous avons tout d’abord démontré par électroantennographie (EAG) que l’huile essentielle de *C. citratus* était une substance active sur les récepteurs olfactifs des antennes de *Stomoxys calcitrans*, par la mise en évidence d’une augmentation significative des réponses EAG à des doses croissantes d’huile essentielle. Des tests de choix réalisés en cage de vol avec des stomoxes ayant à disposition deux supports imprégnés de sang, l’un ayant été traité avec de l’huile essentielle, montrent que les stomoxes (*n* = 24) ont passé significativement plus de temps dans la zone non traitée (valeur médiane = 218,4 s) que dans la zone traitée (valeur médiane = 63,7 s). Aucun stomoxe ne s’est nourri sur le support traité alors que neuf stomoxes se sont nourris sur le support non traité. Ces résultats suggèrent que l’huile essentielle de *C. citratus* pourrait être utilisée comme répulsif contre les stomoxes. Des études complémentaires sont nécessaires pour confirmer ses effets répulsifs et anti-gorgement.

## Introduction

The stable fly *Stomoxys calcitrans* L. is among the most damaging arthropod pest of livestock worldwide [8, 15, 23], with a high economic impact on dairy and beef cattle production [3, 27, 39]. It is also a potential mechanical vector of animal pathogens such as equine infectious anemia virus, *Trypanosoma evansi*, and *Besnoitia besnoiti* [7, 9, 19]. Control of stable fly populations includes various methods, such as chemical control (pesticides and repellents), cultural control (sanitation), mechanical control (trapping devices), and biological control (parasitoids and entomopathogenic fungi) [9, 20]. The best approach is the simultaneous use of several methods in an integrated pest-management program [26]. Management of adult flies is accomplished mainly with topical insecticides, applied directly to animals. However, continued or repeated use of conventional insecticides often results in the development of resistance and fosters serious human health and environmental concerns [13, 42]. Populations of *S. calcitrans* resistant to pyrethroids and/or organophosphates have already been described in North America and in Europe [4, 21, 31, 34]. As a result, there have been increased research efforts for natural and environmentally friendly repellents, particularly those based on essential oils [38]. Several plant-based repellents, such as citronella oil, eucalyptus oil, catnip oil, and zanthoxylum oil, have previously been tested against stable flies and have shown a reduction in attraction and in feeding [1, 13, 40, 43]. These repellents can be applied topically on animals or in livestock barns [13]. The first study demonstrating the potential application of a plant-based repellent was conducted by Zhu et al. [44], in which wax-based catnip pellets spread in the manure/soil areas of cattle feedlots resulted in over 99% repellency of stable flies.

Lemongrass oil is the essential oil obtained from the aerial parts of *Cymbopogon citratus* (DC.) Stapf., Poaceae [29]. Geranial (*α*-citral) and neral (*β*-citral) are the two main active components of lemongrass oil, but other compounds, such as geraniol and citronellol, which are known repellents, are also present in small amounts [2, 18, 38]. Lemongrass essential oil has previously shown a repellent effect, alone or in combination, against different species of disease-transmitting mosquitoes (Diptera: Culicidae) and the house fly *Musca domestica* L. (Diptera: Muscidae) [16, 25, 30, 37], and is already present in commercially available products [5, 32]. Therefore, our objectives were to verify the sensitivity of antennal receptor cells of *S. calcitrans* to lemongrass oil and to evaluate its repellency against stable flies using a video-tracking system.

## Materials and methods

### Insects

*Stomoxys calcitrans* pupae were obtained from the laboratory colony of the National Veterinary School of Toulouse (Toulouse, France) [35]. Newly emerged flies were not sexed. Males and females were enclosed together in a cotton mesh cage (40 cm W × 25 cm H × 25 cm D) at 24 ± 2 °C with 40–50% relative humidity. Flies were fed with 10% sugar water *ad libitum* and, once a day, with citrated bovine blood. Experiments were conducted with 2–4-day-old flies. Flies were not fed for 24 h prior to each test.

### Electroantennogram recording

Following the method used in the study by Jeanbourquin and Guerin [14], electroantennogram (EAG) recordings from antennae of *S. calcitrans* were made with an EAG recording device (EAG combi probe internal gain ×10, CS-55 stimulus controller and IDAC-2 signal acquisition controller, Syntech, Hilversum, the Netherlands). Recordings were made using electrolyte-filled (0.1 M KCl) glass capillary electrodes (Ø 1.5 mm, 40 mm L), with Ag/AgCl wire (Ø 0.5 mm, 20 mm L) making contact with the recording apparatus. The antenna was maintained in a humidified charcoal-filtered air stream delivered at 14.6 mL/s through a metal tube. Aliquots of pure lemongrass oil (from *C. citratus* DC., citral ~75%, Sigma Aldrich Chemie GmbH, Buchs, Switzerland) were prepared using hexane (95%, Carlo Erba Reagenti, Arese, Italy) at 0.1, 0.01, 0.001, 0.0001 mg/μL. Tested solutions (10 μL) were deposited on a strip of filter paper (20 × 5 mm) placed in a glass Pasteur pipette. The solvent was allowed to evaporate for 15 min before first use. The tip of the pipette was connected to the metal tube, and the test stimulus was delivered to the antenna using an air pulse (20 mL/s for 0.6 s). Stimuli were released successively in random order at 90-s intervals to avoid receptor saturation. Octenol (1-octen-3-ol, 98%, Sigma Aldrich Chemie GmbH, Buchs, Switzerland) was used as a positive control and hexane was used as a negative control. Differences in EAG responses were evaluated using a Wilcoxon signed-rank test.

### Bioassays

**Figure 1.**
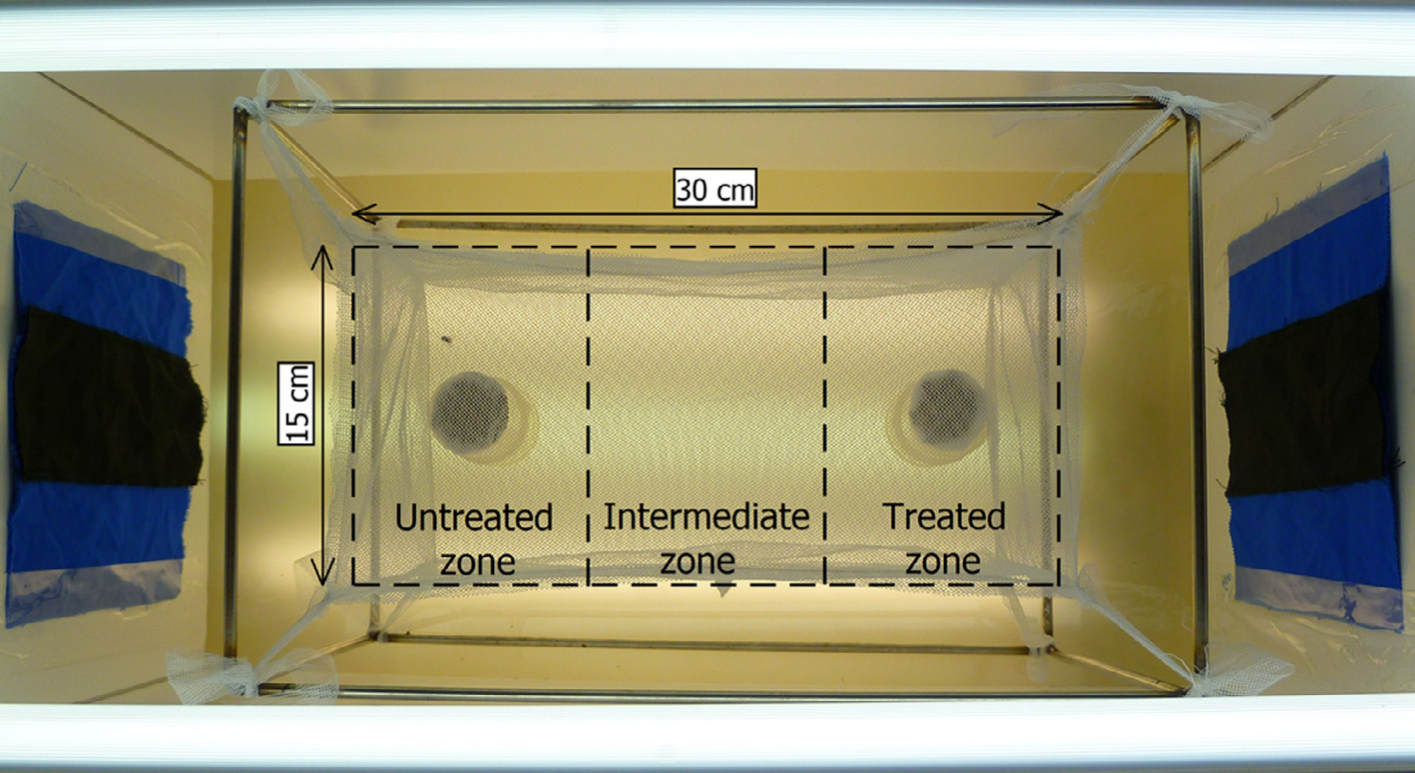
Aerial view of the system for spatial repellency bioassays. The screen cage was made of mosquito netting suspended on a metal frame and surrounded by white foam board with blue and black fabric on each side. Two blood-soaked sanitary pads were set under the cage: one, impregnated with lemongrass oil, was placed in the treated zone, and the other, impregnated with hexane, was placed in the untreated zone. For further details see Materials and Methods section.

To observe the flight behavior of stable flies, we used a screen cage (30 cm W × 15 cm H × 15 cm D) made of polyester mosquito netting suspended on a metal frame (Figure 1). A small hole in the middle of one side of the cage was sealed with a piece of cotton wool and was used to allow the release of one fly at a time into the cage. The cage was surrounded by a shield of white foam board to prevent optical stimulation of the flies. To stimulate the fly to move in the cage, pieces of blue and black fabric (SuperMaine 300 g cotton/polyester 65/35%; TDV industries, Laval, France), commonly used to attract biting flies, were hung on each side of the foam board [17, 24]. Illumination was provided by fluorescent tubes (frequency 50 Hz) placed below and above the screen cage. The light level in the middle of the cage was about 4600–5000 lux.

One fly was released into the cage 15 min before the test. Bioassays were conducted using male and female stable flies during the daytime at ambient laboratory temperatures of 22–26 °C and 40–50% relative humidity. The bioassays consisted of feeding-choice tests in which the fly had access to two blood sources, one of which was treated with lemongrass oil. Citrated bovine blood (1.5 mL), previously heated at 45 °C, was placed on two sanitary pads (Ø 4 cm) from which we removed the outer layer. The outer layer of one pad was impregnated with 100 μL of lemongrass oil solution at 0.1 mg/μL, and the other outer layer with 100 μL of hexane. When the solvent had evaporated, each outer layer was repositioned on top of one of the blood-soaked sanitary pads, which were placed just under the cage floor, 20 cm apart. Fly movement was recorded using a Digital Video Camera Recorder (DCR-SR21E; Sony, Japan) set 1 m above the center of the cage. The behavior of the fly was then recorded during a 10-min period. We tested 4–6 flies each day; the behavior of 24 flies was included in this study. The room was ventilated for at least 30 min between each test, and a new screen cage was used for successive flies. The positions of the pad treated with lemongrass oil and the untreated pad were inversed each time. The cages were cleaned every day by soaking them in a 2% solution of Decon 90 (Decon Laboratories Limited, Sussex, England) for 12 h.

### Video analysis

**Figure 2.**
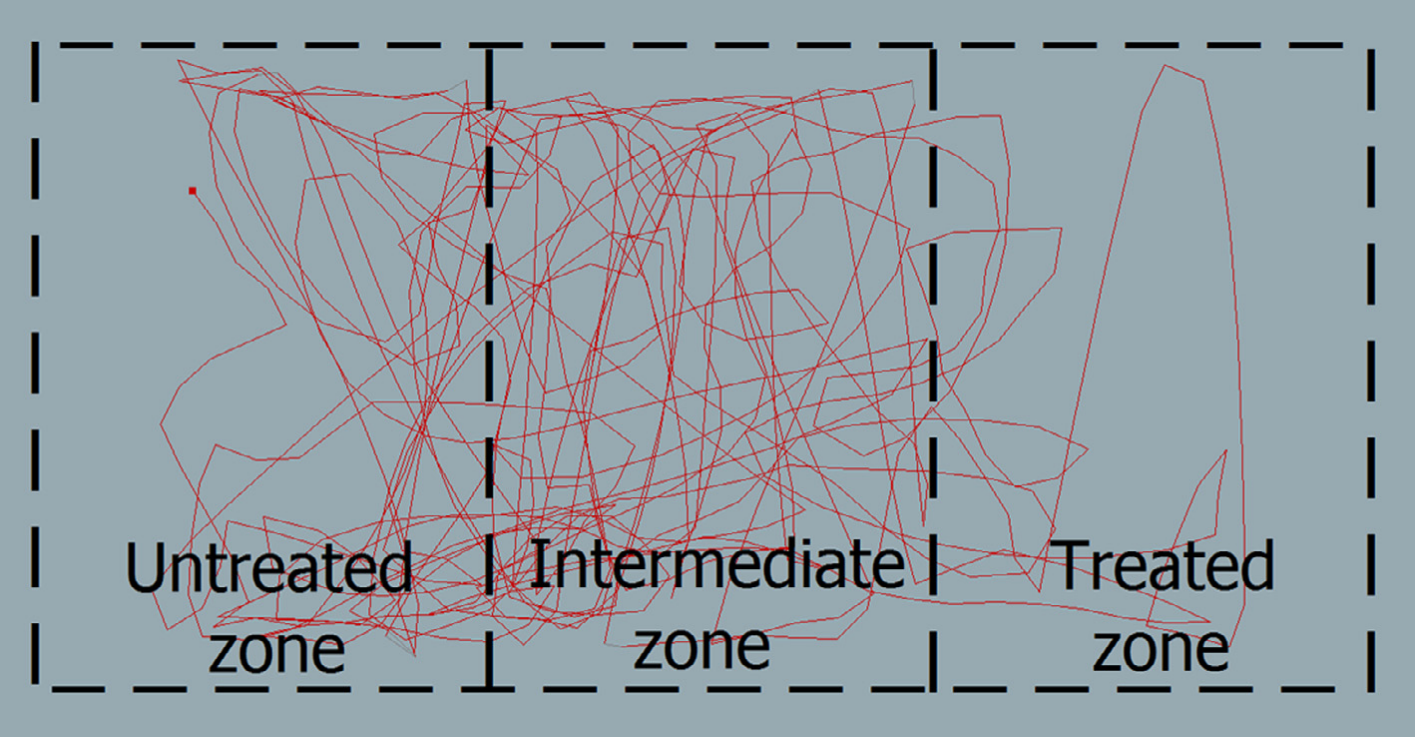
Track showing the 10-min recording of a stable fly in the bioassay cage divided into three zones: the untreated zone, the intermediate zone, and the treated zone.

The video records of fly movement were analyzed using EthoVison XT (v. 8.0; Noldus Information Technology, Wageningen, the Netherlands) [28]. The cage was defined as an arena (30 × 15 cm) divided into three zones (each 10 × 15 cm): untreated, intermediate, and treated (Figure 1). Movement was recorded at 25 video frames per second, and the fly was tracked by dynamic subtraction (Figure 2). In this method, the program compares each sampled image with a reference image that is updated regularly. Image processing algorithms are applied to detect the fly against the background and to extract relevant image features. During data acquisition, EthoVision displays the live video image, tracking statistics (elapsed time, number of samples), and the *x*, *y* co-ordinates of the fly [28]. Several parameters were calculated: the distance moved (in centimeters), the total time spent in each zone (in seconds), the time spent in movement (in seconds), and the mean velocity (centimeters per second). “Moving” and “not moving” were defined with thresholds at 1 and 0.9 cm/s. A comparison between males and females was made with the non-parametric Mann-Whitney test for independent samples. Comparisons of flight parameters between the treated zone and the untreated zone were made with the non-parametric Wilcoxon signed-rank test for two samples of univariate data. All analyses were performed using PAST version 2.12 [12].

### Lemongrass oil volatiles

To estimate the diffusion of lemongrass oil volatiles in the bioassay cage, we compared the atmospheric concentrations of neral and geranial, its most abundant constituents. To accomplish this, three 65 μm Polydimethylsiloxane-Divinylbenzene (PDMS-DVB) fibers (Supelco, Sigma-Aldrich, Bellefonte, PA, USA) were conditioned in the inlet of a gas chromatograph (GC) held at 250 °C for 5 min before sampling. The SPME holders were exposed in the cage for 10 min at three positions. One SPME fiber was positioned 10 cm above each of the two blood-soaked sanitary pads, and another was positioned in the middle of the cage. Relative concentrations of volatile samples were analyzed in a GC-mass spectrometry (MS; Shimadzu QP2010plus, Shimadzu Scientific Instruments, Kyoto, Japan), using helium as the carrier gas (1 mL/min). Samples were injected in splitless mode. The temperature program for GC analyses was 40 °C for 5 min, 5 °C/min to 220 °C, and 10 °C/min to 250 °C.

## Results and discussion

**Figure 3.**
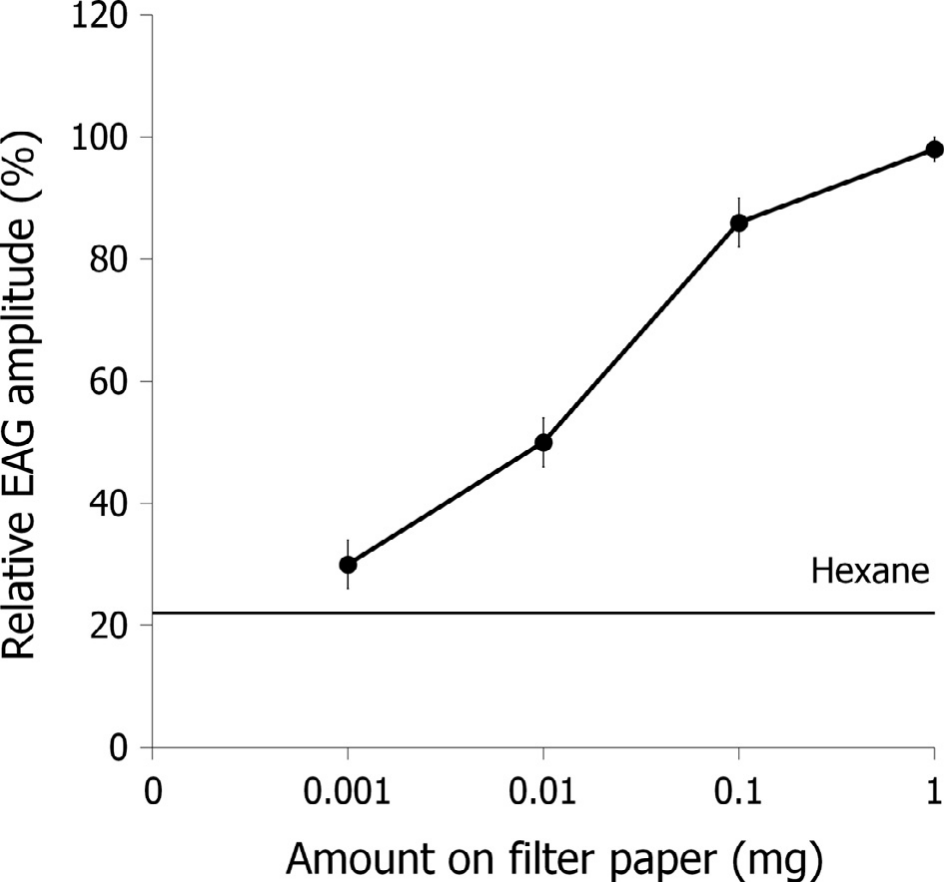
Mean relative EAG amplitudes recorded from *Stomoxys calcitrans* antennae (*n* = 7) stimulated with lemongrass essential oil at doses of 0.001 mg, 0.01 mg, 0.1 mg, and 1 mg. Hexane was used as negative control. EAG amplitudes are relative to the value of 100% for octenol at 1 mg in the stimulus syringe. Differences in EAG amplitudes were evaluated using the Wilcoxon signed-rank test. Significant differences are indicated by different letters (*p* ≤ 0.05).

Our investigation showed that *S. calcitrans* EAG amplitudes increased significantly in a dose-dependent fashion with increasing doses of lemongrass oil in the stimulus pipette. The mean EAG amplitude elicited by each dose (0.001 mg: 2.06 ± 0.37 mV; 0.01 mg: 3.37 ± 0.47 mV; 0.1 mg: 5.80 ± 0.67 mV; 1 mg: 6.50 ± 0.57 mV) was significantly greater than that elicited by hexane (1.46 ± 0.29 mV) (Figure 3) and there was no significant difference between lemongrass oil and the octenol at 1 mg on filter paper (6.64 ± 0.55 mV). Octenol is a very strong chemostimulant for *S. calcitrans* antennae [36, 41] and a good attractant in the field [11]. The study by Zhu *et al.* [44] was the first to report that stable fly antennae are also capable of detecting repellents such as catnip oil. In our study, EAG responses to lemongrass oil at 10 μg (~3350 μV) were nearly five times higher than the EAG responses to the same amount of catnip oil (~700 μV recorded by Zhu *et al.* [44]). These results indicate that lemongrass oil is a strong stimulant for the olfactory receptor cells of *S. calcitrans* and thus a suitable candidate for behavioral tests.

In the bioassays, the amount of lemongrass oil on the treated pad used in all tests was 10 mg. Relative concentrations of neral and geranial in the arena were assessed by the height of their peaks in mass chromatograms to reveal a 12-fold decrease in the atmospheric concentration of lemongrass oil between the treated and untreated pads. It should be noted, however, that this measurement was taken in the absence of a fly. The air flow induced by the flight activity of a fly in the cage might partially disturb this ratio during a test.

**Figure 4.**
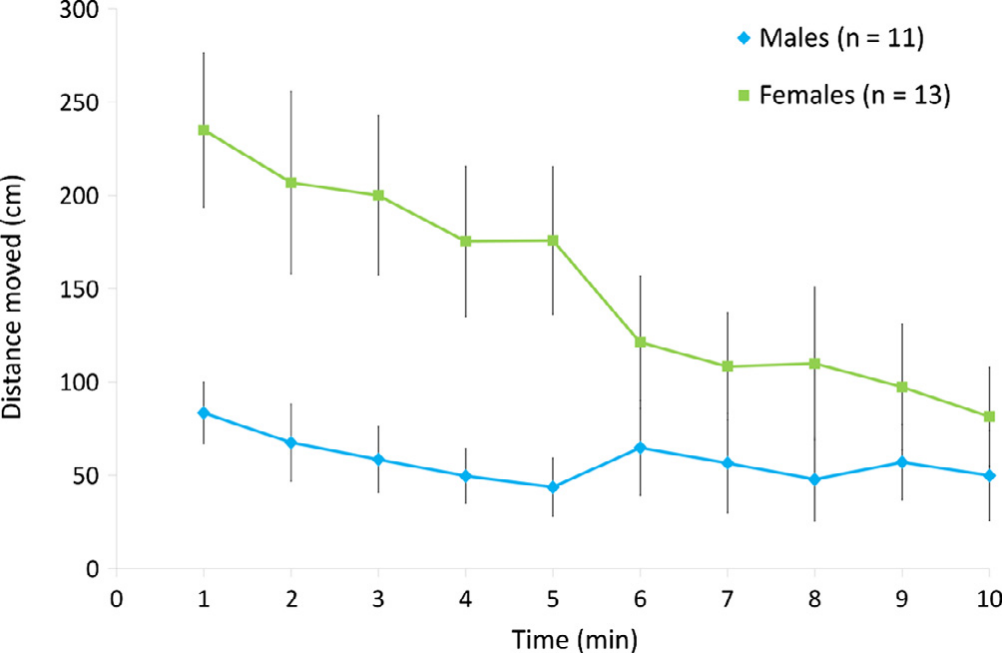
Time-course (mean ± *SE*) of the distance moved by stable fly males and females for 10 min following release into a bioassay cage. Curves represent the distance moved for each successive 1-min period during 10-min recordings.

We tested 11 males and 13 females in the bioassay cage (Table 1). First, we compared the flight activity between the two sexes. The distance moved is considered to be the main indicator of the activity level of a fly [22]. At the beginning, females were more active than males (in terms of time spent in movement and velocity) (Figure 4). Over the duration of the 10-min recordings, the distance moved by females gradually decreased to reach a level similar to males. This decrease in movement might have been due to exposure to lemongrass oil, or simply to acclimation to the bioassay cage. This is an open question as no tests were conducted without a treated pad. However, locomotor activity was sexually distinct, as has been observed in fruit flies, *Drosophila melanogaster* [10].

**Table 1.** Comparison of the flight activity of male and female stable flies (Mann-Whitney *U* test), and comparison of the behavior of flies (both sexes) between the zone treated with lemongrass oil and the untreated zone (Wilcoxon *W* signed-rank test).

	*N*	Median value	Percentiles	Test
Time spent in movement (s)				
Males	11	**95.7**	**38.1–148.8**	***U* = 34**
Females	13	**144.8**	**110.8–177**	***p* = 0.030**
Velocity (cm/s)				
Males	11	**6.9**	**5.7–7.7**	***U* = 10**
Females	13	**15**	**10.4–16.9**	***p* = 0.0001**
Total time (s)				
Treated zone	24	**63.7**	**41–163.7**	***W* = 233**
Untreated zone	24	**218.4**	**94.2–434.2**	***p* = 0.016**
Time spent in movement (s)				
Treated zone	24	22.3	11.3–35.8	*W* = 200
Untreated zone	24	30.6	14–54.3	*p* = 0.160
Velocity (cm/s)				
Treated zone	24	9.8	7.2–14	*W* = 182
Untreated zone	24	9.1	6.7–12.2	*p* = 0.371

Data that show significant differences are indicated in bold.

Comparing the behavior of stable flies in the zone treated with lemongrass oil with their behavior in the untreated zone did not reveal any significant differences between the two zones in terms of the time spent in movement or in the mean velocity of movement (Table 1). However, stable flies spent significantly more time flying in the untreated zone than in the treated zone during the tests. Moreover, we observed nine stable flies feeding on the untreated pad, whereas none fed on the treated pad. The attractiveness of the untreated blood-soaked pad versus the treated pad explains the difference in the total time spent between the two zones. These findings suggest that lemongrass oil could be used as a repellent against stable flies. However, further investigations on spatial repellency and feeding deterrence are necessary to demonstrate that lemongrass oil is as effective as catnip oil against stable flies in the field [44]. Video tracking appears to be a useful tool to study insect behavior in response to repellent volatiles [6, 33], especially for flies, which are otherwise difficult to track.
